# Is the risk of ischemic heart disease in women after radiotherapy for breast cancer nowadays still (linearly) associated with the mean heart dose?

**DOI:** 10.2340/1651-226X.2024.34751

**Published:** 2024-04-10

**Authors:** Henk Struikmans, Anna Petoukhova, Joop H.M. Schreur, Mirjam E. Mast, Philip M. Poortmans

**Affiliations:** a Department of Radiation Therapy, Haaglanden Medical Center, Leidschendam, The Netherlands; b Department of Medical Physics, Haaglanden Medical Center, Leidschendam, the Netherlands; c Cardiology Department, Haaglanden Medical Center, Leidschendam, The Netherlands; d Department of Radiation Oncology, Iridium Netwerk, Wilrijk-Antwerp, Belgium; e Antwerp University, Wilrijk-Antwerp, Belgium

**Keywords:** Breast cancer, radiotherapy, mean heart dose, major coronary events

From the US-SEER cancer registries, it appeared that radiation therapy (RT), administered between 1973 and 1982, resulted in an increased mortality rate up to 10–20 years after treatment for left-sided breast cancer (BC) patients [1]. Furthermore, Darby et al. reported that major cardiac events (MCE) incidence rates increased linearly with mean heart dose (MHD) by 7.4% relative per Gray [2]. Similar results were found in two Dutch and one Danish study [3–5]. Thereafter, MHD is used as a normative planning parameter in BC-RT in daily practice as well as in studies [6–8]. Recently, though, the presence of a dose–effect relationship was not confirmed [9, 10]. Therefore, we decided to evaluate whether a linear association between MHD and MCE-incidence rates still applies and noted:

1. The reported ‘relative excessive risks relative per Gy’ for these six studies varied substantially (0%–19%) [2, 3, 4, 5, 9];
2. In the Darby study, analyses were based on ‘a standard patient’ and in early cases only analogue RT-charts, to estimate dose levels, were available, negatively impacting the reliability of their findings [2, 3, 5];
3. RT details differed or were absent. In contrast to the older studies, in the more recent studies, the majority of patients were treated after 2000 [4, 9, 10]. Whereas after 2000, more homogeneous and conformal 3D dose calculation and delivery was feasible [11, 12];
4. van den Bogaard et al. concluded that their multivariable normal tissue complication probability model, predicting the occurrence of an acute coronary event, included the 5Gy left ventricle volume, but not MHD [4];
5. Age-restrictions as well as countries of residence differed, while age-standardized prevalence of coronary vascular diseases as well as death rates from ischemic heart disease for women vary substantially between European countries, impacting the correct interpretation of these studies [13–15];
6. Only relative risks were provided, whereas clinical relevance is determined also by absolute figures;
7. Information about tobacco use was unknown in >50% of cases [2] and not considered in two other studies [5, 9]. This is relevant since tobacco use is associated with an increased risk of developing MCEs after BC-RT [16–18]. For the combination of tobacco use and BC-RT, even a synergistically increased rate of fatal myocardial infarction was noted [16].

Subsequently, we investigated results of (more recent) phase-III trials, because its prospective design ensures that the reliability of findings of phase-III trials is not hampered by observed differences and methodological flaws. And to confirm whether the reported higher MHD values for locoregional RT, including the internal mammary chain (IMC) [11, 12, 19, 20] coincide with significantly higher MCE-incidence rates. To summarise, firstly the DBCG-82bc trial (*n* = 3,083), after 30-year follow-up, reported no significant excess deaths of ischemic heart disease [21]. Patients were randomized (1982–1990) between yes or no postoperative RT to the chest wall and nodal regions (including IMC). Secondly, in 2023, the meta-analysis of the ‘Early Breast Cancer Trialists’ Collaborative Group’, evaluating the role of regional lymph node irradiation (RNI) in 16 phase-III trials (*n* = 14,324), was reported. With 15 years of follow-up, no increased non-breast cancer-related mortality (including cardiac deaths) was noted in the eight more recent trials (*n* = 12,167), in which patients were treated from 1989 till 2008 [22]. Thirdly, in the EORTC 22922-10925 trial (1996–2004), 4,004 patients were randomized between irradiation of the ipsilateral IMC and ipsilateral medio-supraclavicular chain or not [23]. Special attention was given to prospectively assess incidences of late side effects. After 15.7 years, cardiac death rates were identical in both arms (1.4%). No differences were found between left-sided versus right-sided cases. While cumulative incidence rates for any heart disease after nodal RT showed a limited increase of 1.7%, no increased incidence rates of cross-sectionally registered cardiac diseases, with a score of 2 or higher (CTC-AE 2, assessed every 5 years after treatment), were seen. Moreover, a RT-planning study using a fractionation scheme of 40.05Gy/15fr and representing >75% (concerning irradiation technique) of the participants of the EORTC 10925-22922 trial, revealed that the mean of the MHD values after left-sided whole breast RT and IMC-RT, when compared to that after whole breast RT only, was 13.9 Gy higher (Figure 1A) [19].

**Figure 1:**
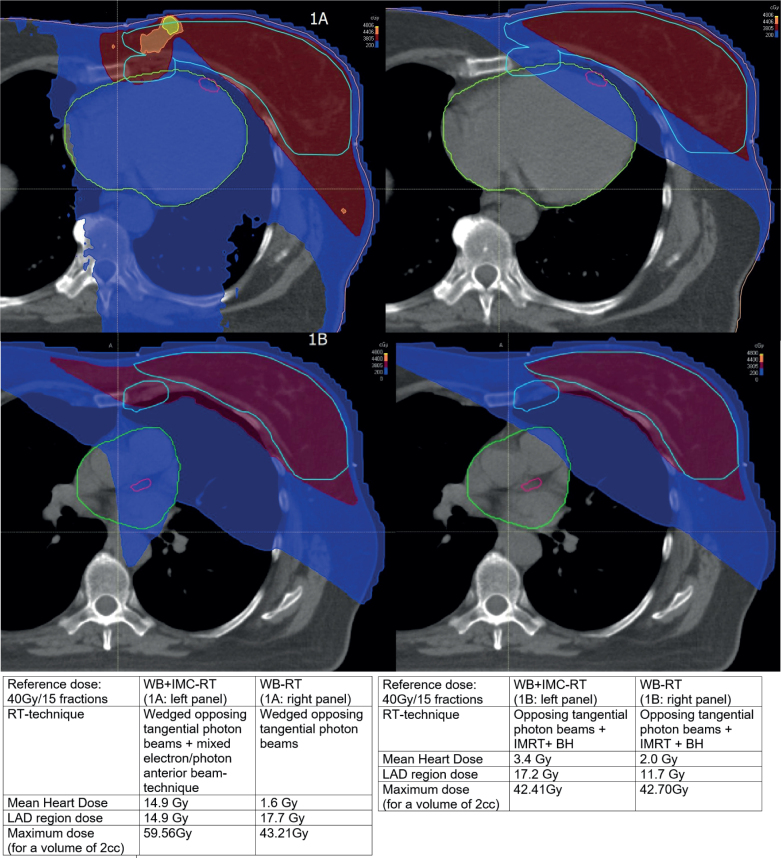
(A) Central plane of a left-sided RT-treatment plan in FB*. Left panel: WB**+IMC***-RT. Right panel: WB-RT. *FB: free breathing; **WB: whole breast; ***IMC: internal mammary chain. Light blue line: PTV*-GBT** and PTV-IMC. Pink line: LAD*** region. Green line: Heart (including pericardium). *PTV: planning target volume; **GBT: glandular breast tissue; ***LAD: left anterior descending coronary artery. Dark red area: 95% reference dose. Dark blue area: 5% reference dose. Orange area: 110% reference dose. Yellow area: 120% reference dose. (B) Central plane of a left-sided RT-treatment plan in BH*. Left panel: WB**+IMC***-RT. Right panel: WB-RT. *BH: breath-hold; **WB: whole breast; ***IMC: internal mammary chain. Technique: Hybrid IMRT*+BH. *IMRT: intensity modulated radiation therapy. Light blue lines: PTV*-GBT** and PTV-IMC. Pink line: LAD*** region. Green line: Heart (including pericardium). *PTV: planning target volume; **GBT: glandular breast tissue; ***LAD: left anterior descending coronary artery. Dark red area: 95% reference dose. Blue area: 5% reference dose. Orange area: 110% reference dose (not applicable). Yellow area: 120% reference dose (not applicable).

This contradicts the findings of Darby et al., predicting an increased MCE-incidence in the treatment arm of this trial of about 100% (13.9 × 7.4%) [2]. According to the results of two similar studies, even more pronounced increased MCE incidences were predicted [4, 5]. The following changes over time should be considered also, between 1970 and 2000, for women in Western Europe, an almost linear decline in death rates, ranging from 65% to 50%, for ischemic heart disease was noted [15]. After 1990, a significant lowering of heart doses in radiotherapy was achieved [11, 12]. More recently, introduction of 3D-treatment planning and, subsequently, intensity-modulated RT (IMRT) and respiratory control enabled even further improved heart sparing [19, 24] (Figure 1A+1B). Another RT-planning study (not considering IMRT), concluded that breath-hold as well as volumetric arc therapy enabled improved heart sparing after nodal RT (including IMC) [20]. After 2008, total RT-doses of 50 Gy/25 fr decreased towards 40 Gy/15 fr [25], resulting in correspondingly decreased mean heart physical doses. Furthermore, according to the results of the ‘Fast-Forward trial’, RT doses for local treatment are decreasing further towards 26Gy/5fr [26]. Radiobiological calculations, though, show that hypofractionation does not increase the probability of RT-associated MCE incidences. Finally, MHD is not a specific tool to predict the incidence of (major) atherosclerotic coronary artery disease, because the dose distribution in the heart is inhomogeneous, especially when using tangential beam-based RT techniques [10, 27].

We conclude that MHD cannot be seen as a reliable tool predicting increased MCE-incidences after BC-RT. Hence, nowadays there is no convincing evidence to support the use of MHD as a normative RT-planning parameter in daily practice nor in studies. The reliability of future findings of studies still using MHD as a normative RT planning parameter is hampered. We state that, in daily practice as well as in studies, cardiac risk assessment in BC-RT is of importance and preferably determined according to reported ‘evidence-based’ guidelines [14]. To predict radiation associated major coronary event incidence rates, we recommend evaluating the relevance of dose-volume relationships for both coronary arteries and left ventricle.

## Data Availability

No specific data were used to compose this letter.
